# Identification the Pathogen Cause a New Apple Leaf Blight in China and Determination the Controlling Efficacy for Five Botanical Fungicides

**DOI:** 10.3390/jof10040255

**Published:** 2024-03-27

**Authors:** Enchen Li, Jia Liu, Shuwu Zhang, Bingliang Xu

**Affiliations:** 1 College of Plant Protection, Gansu Agricultural University, Lanzhou 730070, China; liec188@163.com (E.L.); jiajia7635724@163.com (J.L.); 2 Gansu Provincial Biocontrol Engineering Laboratory of Crop Diseases and Pests, Lanzhou 730070, China; 3 Gansu Provincial Key Laboratory of Arid Land Crop Science, Gansu Agricultural University, Lanzhou 730070, China

**Keywords:** apple, leaf blight, pathogen identification, multi-locus phylogeny, *Alternaria tenuissima*, biological characteristics, botanical fungicides

## Abstract

Alternaria leaf blight has recently been described as an emerging fungal disease of apple trees which is causing the significant damage in the apple-growing areas of Tianshui and Jingning, Gansu, China. In the present study, the pathogen species involved in apple leaf blight and its biological characteristics were identified, and the inhibitory activity of different botanical fungicides against the pathogen was evaluated *in vitro*. Four strains were isolated from the symptomatic areas of necrotic apple leaves, and initially healthy leaves showed similar symptoms to those observed in orchards after inoculation with the ABL2 isolate. The ABL2 isolate was identified as *Alternaria tenuissima* based on the morphological characteristics of its colonies, conidiophores, and conidia, and this was also confirmed by multi-gene sequence (ITS, OPA10-2, *Alta-1*, and *endoPG*) analysis and phylogenic analysis. The optimum temperature, pH, carbon source, and nitrogen source for the growth of *A. tenuissima* mycelia were 28 °C, 6–7, soluble starch, and soy flour, respectively. In addition, the botanical fungicide eugenol exhibited the highest inhibitory effect on the mycelial growth and conidia germination of *A. tenuissima*, and the median effective concentration (EC_50_) values were 0.826 and 0.755 μg/mL, respectively. The protective and curative efficacy of eugenol were 86.85% and 76.94% after inoculation in detached apple leaves at a concentration of 4 μg/mL. Our research provides new insights into the control of apple leaf blight disease by applying botanical fungicides.

## 1. Introduction

Apples are one of the most widely cultivated and consumed fruits in the world and can look back on a history of cultivation spanning thousands of years [1]. The size of the areas undertaking the cultivation and output of apples in China has taken a leading position in the world in recent years [2] and is increasing year on year due to their high economic and nutritional value. However, apple leaf diseases can cause significant production and economic losses, as well as a decline in the quality and quantity of fruit production [3]. Alternaria leaf blotch, Marssonina leaf blotch, Glomerella leaf spot, and powdery mildew have been the most common and serious apple leaf diseases in recent years, and their causal pathogens have been identified as *Alternaria mali*, *Marssonina coronaria*, *Glomerella cingulata*, and *Podosphaera leucotricha*, respectively [4,5,6,7,8,9]. Alternaria leaf blotch is related to different taxa of *Alternaria*, which is prevalent in major apple-producing regions around the world [10,11]. In some susceptible varieties, Alternaria leaf blotch can cause up to 80% of early tree defoliation, which impairs the vitality of the trees and drastically reduces fruit yields [12,13,14]. Previous studies have shown that *Alternaria* spp. on apples mainly causes two different diseases: Alternaria leaf (fruit) spot and mildew core disease. Since 2019, we have discovered a new apple leaf disease in many apple-producing areas in Gansu Province, China. The symptoms of this disease differ from those previously reported for Alternaria leaf blotch. The pathogen of this disease spreads rapidly and has a higher virulence, leading to rapid drying and shedding of infected leaves within a short period, resulting in lower fruit quality and yield. However, the pathogen that causes this disease is still unknown.

Accurate identification of the pathogen of apple leaf blight is crucial for the epidemiology and management of the disease. Although the conidia of the genus *Alternaria* are distinct and easy to recognize, due to their highly similar morphological characteristics, many species of *Alternaria* have high isolate variability even within the same species [15,16,17]. The morphological characteristics of *Alternaria* species can change with the changes of environmental conditions [18]. Therefore, it is difficult to identify species of *Alternaria* based solely on morphology. Phylogenetic analysis based on polygenic loci has been used to identify *Alternaria* species as a useful supplement to morphological methods [19]. Several gene regions, such as the internal transcribed spacer (ITS), Alternaria major allergen 1 (*Alta-1*), endopolygalacturonase (*endoPG*), and anonymous noncoding gene region (OPA10-2), have proven useful in evaluating the phylogenetic interspecies relationships of *Alternaria* species [20].

Currently, synthetic fungicides are mainly used for the prevention and control of Alternaria leaf blotch disease in apples caused by *Alternaria* spp., and the effective synthetic agents are mainly tebuconazole, mancozeb, thiophanate methyl, carbendazim, and other fungicides [21]. However, a series of environmental problems and an increase in the risk of pathogen resistance have occurred due to the long-term use of synthetic fungicides, as well as an increase in production costs and a deterioration in fruit quality [22]. The development and use of environmentally friendly bio-fungicides has become a trend in the sustainable development of modern agriculture, with people’s increasing attention to environmental protection. Botanical fungicides have been used as alternative remedies to treat plant diseases due to their being effective, selective, biodegradable, and less toxic to the environment [23]. Plant extracts, essential oils, etc., are some of the botanical fungicides that have been shown to exert biological activity against plant fungal pathogens [24,25]. In recent years, many studies have reported on the antifungal activities of botanical fungicides or plant extracts against different plant Alternaria leaf blight pathogens, such as the botanicals of drake (*Melia azedarach*), which has a mycelial growth inhibition rate of 63.52% against Alternaria leaf blight of tomato caused by *A. solani* [26]. Methanolic leaf extracts of *Elettaria cardamomum*, *Syzygium aromaticum*, and *Curcuma longa* and root extract of *Parthenium hysterophorus* showed up to 100% inhibition of *A. solani* (potato) [27]. In sunflower, botanicals (Neem and Karanj) can significantly inhibit the growth of mycelia and disease intensity of Alternaria blight (*A. helianthi*) [28]. In contrast, few studies have focused on the antifungal activities of botanical fungicides against apple leaf diseases caused by *Alternaria* spp. [29].

There are few available data on the phenotypic characters and phylogenetic interspecies relationships of pathogen causing leaf blight disease on apples in China. In addition, understanding the biological characteristics of the pathogen and screening environmentally friendly fungicides may provide valuable data for formulating disease management strategies. Therefore, the purposes of this research were to: (i) isolate and identify the pathogen causing apple leaf blight in Gansu Province; (ii) determine the biological characteristics of the pathogen; (iii) assess the antifungal activity of five botanical fungicides against the pathogen.

## 2. Materials and Methods

### 2.1. Pathogen Isolation and Purification

One-hundred diseased apple (cultivars, ‘Fuji’ and ‘Gold Delicious’) leaf samples were collected from different locations in Gansu Province in August 2020 and 2021. Several pieces of the tissue at the intersection of disease and health of the diseased apple leaves (5 mm × 5 mm) were disinfected with 75% alcohol for 10 s and 1% sodium hypochlorite (NaOCl) for 3 min, and then rinsed with sterile water for three times. The sterilized leaf sections were dried on sterile filter paper and placed onto potato dextrose agar (PDA) plates (5 pieces per dish), and then the plates were incubated at 28 °C in the dark for 5 days. For each isolate, single-spore isolate was obtained from the sub-cultured fungal colonies using the dilution plate technique [30]. Purified single-spore isolates were obtained by sub-culturing three times on PDA at 28 °C with a light/dark (12/12 h) cycle for 5 days, and then stored at 4 °C for further study. 

### 2.2. Pathogenicity Testing

Pathogenicity testing was conducted according to the method described by Li, et al. [31], with minimal modification. Healthy leaves of the same species of ‘Fuji’ were collected from Jingning, Gansu Province, and used for pathogenicity testing. Asymptomatic and similar-sized leaves were disinfected with 75% ethanol for 1 min, rinsed three times with sterile water, and air-dried. The surface and petiole of the leaves were wounded with sterile needles, and mycelial plugs (5 mm diameter) were taken from the margin of 5-day-old colonies of the isolates and inoculated on the wounds, respectively. Leaves inoculated with agar plugs of the same diameter served as the control. Twenty leaves were used for each isolate, and the test was repeated twice. The inoculated apple leaves were then kept at 28 °C with a 16 h/8 h light/dark cycle and 80% relative humidity for determining the pathogenicity of each isolate. The disease symptoms in the leaves were examined at 10 days after inoculation. Fungal pathogens were re-isolated from the symptomatic tissues of inoculated leaves and compared to the initial inoculation according to morphological characteristics.

### 2.3. Pathogen Identification

#### 2.3.1. Morphological Observations

The morphological characteristics of ABL2 was recorded by inoculating on PDA plates with a light/dark cycle of 12/12 h at 28 °C for 5 days, and then the colony color and characteristics were recorded. The characteristics of conidiophore and conidia were observed by using a Nikon DS-Ri2 microscope, and the size of conidiophore and conidia were measured, each with 50 replications.

#### 2.3.2. DNA Extraction, PCR Amplification

The genomic DNA of the ABL2 isolate was extracted using an E.Z.N.A. ^®^ High Performance (HP) Fungal DNA Kit (OMEGA Bio-Tek, Norcross, GA, USA) and stored at -20°C. Four regions, the internal transcribed spacer (ITS), anonymous noncoding region (OPA10-2), Alternaria major allergen 1 (*Alta-1*), and endopolygalacturonase (*endoPG*), were used for the phylogenetic analysis of ABL2. The primers used in this study (Table 1) were synthesized by Sangon Biotech Co., Ltd. (Shanghai, China). Each polymerase chain reaction (PCR) contained (25 μL) 1 μL genomic DNA (50 ng), 10 μL of ddH_2_O, 1 μL of each primer (10 μM), and 12 μL of Taq PCR Mastermix (Tiangen Biotech, Beijing, China). PCR amplification was performed using an Eppendorf Mastercycler^®^ Gradient PCR machine (Eppendorf Scientific, Hamburg, Germany). Amplification was performed using the following parameters: pre-denaturation at 94 °C for 4 min, followed by 30 cycles of denaturation at 94 °C for 30 s, annealing (Table 1), extension at 72 °C for 1 min, and a final extension at 72 °C for 10 min.

#### 2.3.3. Phylogenetic Analysis

The PCR products were sent to Sangon Biotech Co., Ltd. (Shanghai, China) for sequencing, and the obtained nucleotide sequences of ITS, *Alta-1*, *endoPG*, and OPA10-2 were deposited in GenBank with the accession numbers. The results were compared with BLAST sequences in the National Center for Biotechnology Information (NCBI) database. For phylogenetic analysis, the homologous *Alternaria* sequences were retrieved from GenBank using the BLASTn program and from a previous study [20] (Table S1). Sequences of each genetic region were aligned to the reference sequences in GenBank by using BioEdit 7.2.6.1 with manual adjustments. Multiple comparisons were performed using MAFF v7.037b software, while conservative region selection was performed using GBLOCKS 0.91b software. Aligned sequences of each of the three genes (*Alta-1*, *endoPG*, and OPA10-2) were combined using Sequence Matrix 1.7.8 software. Phylogenetic trees were constructed using combined sequences from the Clustal W 2.0 multiple alignment output using the GTRCAT nucleotide substitution model and maximum likelihood (ML) method in the MEGA X (version 10.1.7) software [35]. The bootstrap analyses were calculated using 1000 replicates under the same model. The phylogenetic tree was edited and visualized using the online tool tvBOT [36].

### 2.4. Biological Characteristics

#### 2.4.1. Effect of Different Carbon and Nitrogen Sources on Mycelial Growth of the ABL2 Isolate

The medium of Czapek Dox Agar (30 g/L of sucrose, 0.5 g/L of MgSO_4_·7H_2_O, 0.5 g/L of KCl, 3 g/L of NaNO_3_, 0.01 g/L of FeSO_4_, 1 g/L of KH_2_PO_4_, 15 g/L of agar, initial pH value is 6.5) served as base medium for determining the effect of different carbon and nitrogen sources on ABL2 mycelial growth. Sucrose, maltose, fructose, mannitol, dextrose, and soluble starch were selected as the sole carbon sources to replace the carbon source (sucrose) in the Czapek medium with the same amount of addition; soy flour, ammonium nitrate, beef extract, sodium nitrate, peptone, and yeast extract were selected as the sole nitrogen sources to replace the nitrogen source (sucrose) in the Czapek medium with the same addition amount. Mycelial plugs (5 mm diameter) from the margins of 5-day-old ABL2 colonies on PDA were transferred to media with different carbon and nitrogen sources. Each treatment was then placed in an incubator at 28 °C in the dark. The growth of ABL2 was evaluated after 7 days by measuring the colony diameter of each treatment (minus the diameter of the inoculation plug). There were three replicates in each treatment, and the experiment was performed three times. 

#### 2.4.2. Effects of Light Regime, Temperature and Different pH on Mycelial Growth of the ABL2 Isolate

Mycelial plugs (5 mm diameter) from ABL2 the edge of 5-day-old colonies were placed on PDA plates and incubated at 28 °C. The light period was set as follows: full dark, full light, or 12/12 h light/dark. To study the effect of temperature on the radial growth of ABL2, the inoculated PDA plates were placed in the dark at 5 °C, 10 °C, 20 °C, 25 °C, 28 °C, 30 °C, and 35 °C. 

The PDA medium was adjusted to a desired pH value ranging from 3 to 12 in increments of one by using HCl or NaOH (Sigma Chemical Co., St. Louis, MO, USA). The mycelial plugs (5 mm diameter) of each 5-day-old colony were transferred to the center of PDA plates with different pH values and incubated at 28 °C in the dark. 

Each treatment with a specific light exposure time, pH value, and temperature was replicated three times, with each PDA plate considered a replicate. After being inoculated for 7 days at different conditions, the colony diameters (minus the diameter of the inoculation plug) were measured and recorded. Each experiment was repeated three times.

### 2.5. Efficacy of Different Botanical Fungicides against the Mycelial Growth of the ABL2 Isolate

Five commercial formulations botanical fungicides, matrine (Tianjin Hengyuan Weiye Biotechnology Development Co., Ltd., Tianjin, China), eugenol (Shandong Yijia Chemical Technology Co., Ltd., Zaozhuang, China), carvacrol (Lanzhou Shichuang Biotechnology Co., Ltd., Lanzhou, China), ethylicin (Kaifeng Dadi Agrochemical Biotechnology Co., Ltd., Kaifeng, China), and berberine (Hebei Wante Biochemistry Co., Ltd., Shijiazhuang, China), as well as a synthetic fungicide dithianon + pyraclostrobin (Qingdao Star Cropscience Co., Ltd., Qingdao, China), were applied in the present experiment for determining their efficacy against the mycelial growth of ABL2. The concentrations of five botanical fungicides and one synthetic fungicide are listed in Table 2. Mycelial plugs (5 mm diameter) from the margin of 5-day-old colonies of ABL2 were transferred to PDA plates containing different concentrations of fungicides. The colony diameters (minus the diameter of the inoculation plug) were measured by measuring the average diameter in two perpendicular directions at 7 days after incubation at 28 °C PDA plates without fungicide (negative control) and PDA plates with different concentrations of dithianon + pyraclostrobin (positive control) were used as the control. Antifungal activity was expressed in terms of percentage of mycelial growth inhibition (MGI) and calculated according to the following formula: MGI% = [(*a* − *b*)/*a*] × 100, where *a* is ABL2 colony diameter of the negative control and *b* is ABL2 colony diameter in the presence of the fungicides.

The EC_50_ (the concentration which reduced mycelial growth by 50%) values were calculated by regressing the inhibition of radial growth values (%control) against the log_10_ values of the fungicide concentrations [37,38]. These were performed with three replicates per concentration of each agent, and the experiment was performed three times.

### 2.6. Efficacy of Different Botanical Fungicides against ABL2 Spore Germination

For the determination the efficacy of different botanical fungicides against spore germination, a spore suspension of ABL2 was prepared at the concentration of 10^6^ spores/mL after incubation for 7 days on PDA. Then, 40 μL of spore suspension was transferred to sterile slides containing 40 μL of Malt Extract Broth (MEB) with different concentrations of fungicides (Table 2). The MEB supplemented with the same amount of sterile distilled water (negative control) and different concentrations of dithianon + pyraclostrobin (positive control) were used as the controls. Thereafter, the sterile slides were placed on moist filter paper in Petri plates, and incubated at 25 °C for 8 h in dark. The number of spores germinated were determined under microscope using a micrometer. The spore germination rates were recorded when the length of a germ tube was equal to or more than half of the diameter of a spore. The experiment was repeated three times and at least 100 spores of each replicate were observed.

The efficacy of different botanical fungicides inhibited the spore germination were expressed as percent spore germination inhibition (GI) and calculated using the following equation: GI (%) = [(Gc − Gt)/Gc)] × 100, where Gc represents the average spore germination number of the control and Gt represents the average spore germination number treated with the fungicide. The EC_50_ values for ABL2 isolate was calculated by regressing percentage spore germination inhibition against the log_10_ of fungicide concentration. 

### 2.7. Protective and Curative Activity of Eugenol on Detached Apple Leaves

The protective and curative activity of eugenol on apple leaves was determined according to Duan, et al. [39], with some modifications. The surface of healthy apple leaves of the ‘Fuji’ variety was disinfected with 75% ethanol for 1 min, washed with sterile water three times, and then the excess water was removed by sterile filter paper. The soluble eugenol concentrate was diluted to 4 μg/mL with sterile water containing 0.1% Tween 80. The leaf age, growth position, and size of all treatments were similar. For the protective activity assay, the surface and petiole of apple leaves were wounded with a sterilized needle, avoiding the midrib, and sprayed with eugenol (4 μg/mL) until liquid flowed onto the surface. After 24 h, the mycelial plugs (5 mm in diameter) were inoculated from the 5-day-old colony of the ABL2 isolate. For the curative effect assay, the leaves were sprayed with eugenol 24 h after inoculation with the mycelial plugs of ABL2 isolate. Sterile water containing 0.1% Tween 80 was sprayed instead of eugenol as the control. The inoculated apple leaves were then placed at 28°C and 80% RH, under a 16 h photoperiod for disease development. Photos were taken to record the disease status of the leaves, analyze the damage area of the leaves using ImageJ software (version 1.8.0) (http://rsb.info.nih.gov/ij/, accessed on 22 October 2022.) after 10 days, and calculate the disease severity based on the area of lesions on each leaf; the formula is as follows: Disease severity (%) = (Infected tissue area)/(Total tissue area) × 100. The experiment was performed twice with twenty leaves per treatment.

### 2.8. Statistical Data Analysis

Unless otherwise specified, all experiments were conducted at least three times, with three replicates for each treatment. Data are expressed as mean ± SD (standard deviation). Statistical analyses were performed using the SPSS version 26.0 (SPSS Inc., Chicago, IL, USA). All data were tested for normally distributed data and homogeneity of variance before post hoc testing. Differences between different treatments were tested post hoc using Duncan’s multiple comparisons.

## 3. Results

### 3.1. Field Symptoms of Apple Leaf Blight

**Figure 1:**
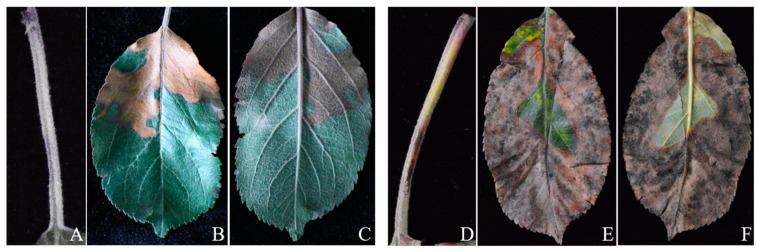
Field disease symptoms of apple leaves. (**A**–**C**) represent the symptoms of leaf blight on the petiole, leaf front, and leaf back of ‘Fuji’. (**D**–**F**) represent the symptoms of leaf blight on the petiole, leaf front, and leaf back of ‘Golden Delicious’, respectively.

During the period of July to October of 2020, apple leaf blight disease was observed on the foliage in several apple orchards (‘Fuji’ and ‘Golden Delicious’) in Pingliang and Tianshui City, Gansu Province. At the initial stage, brownish lesions appear on the petiole in ‘Fuji’ (Figure 1A) and ‘Golden Delicious’ (Figure 1D), and then spread from the petiole along the leaf margin to the inside of the leaf, forming stripes or irregular shapes of lesions (Figure 1B,C,E). As the disease progresses, the lesions rapidly expand inward along the leaf margin to the entire leaf, causing the leaf to dry up and easily fall off (Figure 1E). A grayish black mold layer appears on the lesions on the back of the leaf under high humidity (Figure 1F). The disease generally began in early June and peaked in July and August, causing a large number of premature defoliations of apple trees and ultimately a decline in apple production. 

### 3.2. Pathogen Isolation and Pathogenicity Testing

**Figure 2:**
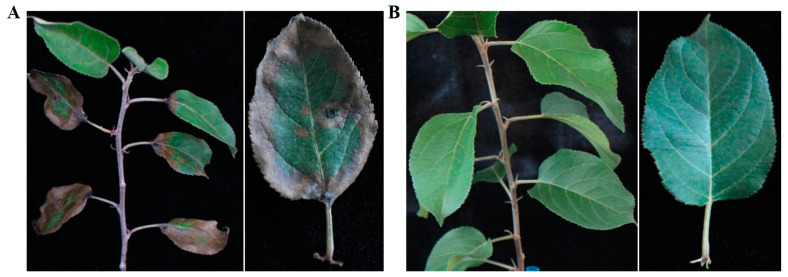
The symptoms of apple leaf blight caused by artificial inoculation. (**A**) Infected symptoms on detached wounded leaves of apple cultivar ‘Fuji’ 10 days after artificial infection with ABL2; (**B**) inoculated with PDA plugs (control).

Eighty-five isolates were isolated from the collected samples of ‘Fuji’ leaves (80) and ‘Golden Delicious’ leaves (20), which were classified into four types. Among them, *Alternaria* spp. was the dominant type, with a total of 73 isolates. The *Alternaria* species were primary classified as ABL1 (4 isolates from ‘Fuji’ and 2 isolates from ‘Golden Delicious’), ABL2 (48 isolates from ‘Fuji’ and 8 isolates from ‘Golden Delicious’), ABL3 (3 isolates from ‘Fuji’), and ABL4 (5 isolates from ‘Fuji’ and 3 isolates from ‘Golden Delicious’) according to the colonies and conidial morphology. The pathogenicity of four types of isolates was determined by needle-puncture method. The results showed that 10 days after inoculating ABL2, apple leaves developed lesions with symptoms similar to those initially observed in the field (Figure 2A), while control leaves remained healthy (Figure 2B). The fungus was re-isolated from diseased leaf tissue using the above method, and microscopic observation showed that its colony morphology and conidial structure were similar to ABL2. However, no obvious symptoms appeared after inoculation with the other types of isolates. The isolate ABL2 was determined to be the causal agent of apple leaf blight according to Koch’s postulates.

### 3.3. Morphological Characteristics of Cultures and Sporulation

**Figure 3:**
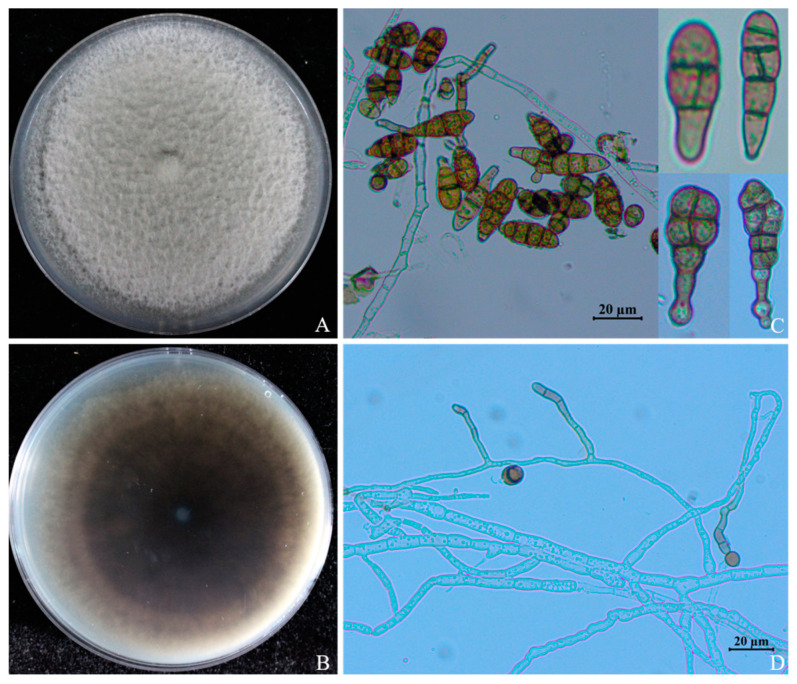
Morphological characteristics of *Alternaria* isolate ABL2 on PDA. (**A**) Colony front on PDA medium; (**B**) Colony reverse on PDA medium; (**C**) Conidia; (**D**) Conidiophore.

The colonies in the pre-growth stage of ABL2 on PDA were grayish white; in the later stages, the color of the colony center gradually deepened to green, the shape was slightly convex, the surface hyphae was dense and tapered, the edges were neat, and the hyphal layer was thick (Figure 3A,B). Conidiophores solitary or terminally erecting from hyphae, slightly curved, pale brown to brown, commonly 10 to 60 × 4 to 6 μm with 1–5 septa (Figure 3D). The conidia had smooth surfaces, were brown or dark brown, typically obclavate or club-shaped, multi-septate, and had 1–5 transverse septa and 0~3 longitudinal septa (Figure 3C). Conidia ranged from 7.5 to 32.6 × 3.10 to 15.71 μm in size (*n* = 50). 

### 3.4. Molecular Identification

The genomic DNA of ABL2 was extracted and used as a template. The PCR product sequence of the isolate have been submitted to GenBank (accession numbers: MZ150772 for ITS, MZ222271 for *Alta-1*, MZ222269 for *endoPG*, and MZ222272 for OPA10-2). The ITS sequence of ABL2 were BLASTn similarity aligned in NCBI, and the online alignment results showed that the sequences of the isolate ABL2 had more than 99.30% identity for *A. tenuissima* and *A. alternata* (MN856355, MN822659, MH447270.1, et al.), but the species of *Alternaria* could not be confirmed by the sequence of ITS. The *Alta-1* gene region sequence of *A. tenuissima* was 99.41% similar to the reference sequences (accession numbers: MW016006). The regional sequence of *endoPG* was compared with the reference sequence of *A. tenuissima* in GenBank, and the results showed 99.79% similarity with the reference sequence (accession numbers: MW016002). The OPA10-2 gene region sequence had 100% similarity with reference sequence of the same species (accession numbers EF503988).

**Figure 4:**
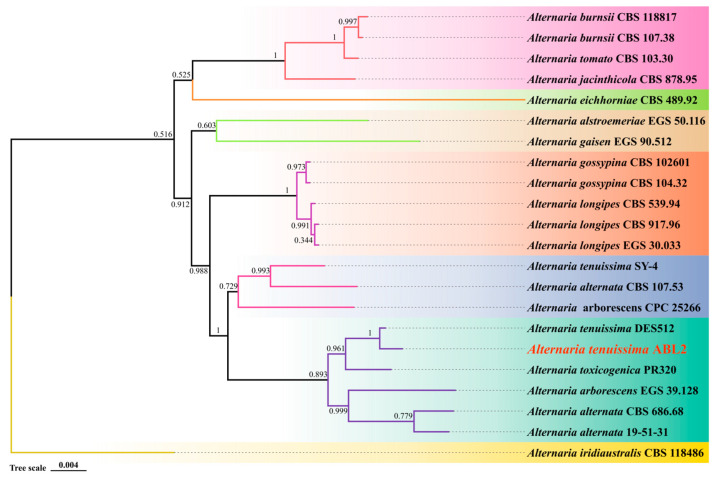
Maximum likelihood (ML) phylogram of the present *Alternaria* isolate ABL2 (red words) based on combined *Alta-1*, *endoPG*, and OPA10-2 genes sequence. *Alternaria iridiaustralis* CBS 118486 was selected as outgroup. Colors indicate clades seen on the concatenated phylogram of the three loci. The numbers beside all branches represent bootstrap values generated from 1000 replicates.

The three genes of *Alta-1*, *endoPG*, and OPA10-2 were connected in series by SequenceMatrix 1.7.8 software in the following order: *Alta-1*, *endoPG*, and OPA10-2. The phylogenetic analysis was conducted for the combined data set by ML. The results indicated that the sequence of each gene of isolate ABL2 was identical to *Alternaria tenuissima*, and they were grouped together with a bootstrap value of 1 (Figure 4). Combined with the morphological characteristics of the pathogenic isolate ABL2, the pathogen was finally identified as *A. tenuissima*.

### 3.5. Biological Characteristics

**Figure 5:**
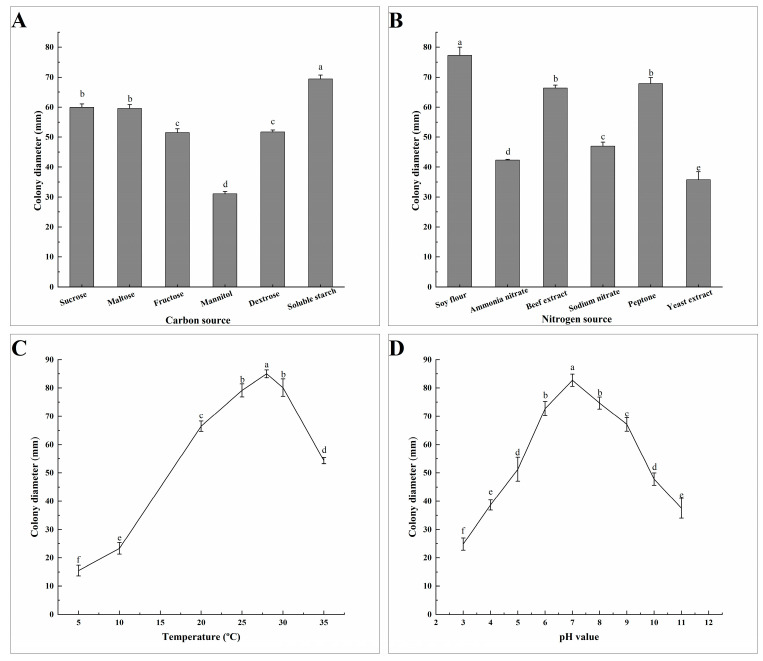
Effects of carbon source (**A**), nitrogen source (**B**), temperature (**C**) and pH (**D**) on the mycelial growth of *A. tenuissima* ABL2. Effect of carbon source on the mycelial growth of *A. tenuissima* ABL2. Bars represent the standard deviations of the means. Means followed by the different letters on the top of each column are significantly different at the *p* < 0.05 level of confidence according to Duncan’s multiple range test.

Different carbon sources, nitrogen sources, pH, and temperatures have varying degrees of influence on the mycelial growth of *A. tenuissima* ABL2. After inoculating on different carbon source media for 7 days, colony morphology and diameter were observed and measured. The results showed that the pathogen grew fastest on the soluble starch medium, with a diameter of 69.46 mm and thick, light brown colonies, which was a significant difference from the other carbon sources. The growth rate was the slowest on the mannitol medium, with a colony diameter of 31.08 mm and thin, milky white colonies (Figure 5A). The pathogen grew faster on soy flour, peptone, and beef extract after being cultured on different nitrogen media for 7 days. The mycelia of ABL2 grew fastest when soy flour was used as the nitrogen source, and the colonies were thick and dark brown with a diameter of 77.28 mm. The mycelia grew slowly when yeast extract was used as the nitrogen source (Figure 5B). The results showed that ABL2 had the highest utilization rates of soluble starch and soy flour.

There was no significant difference in colony growth of ABL2 under different light treatments after 7 days. Under different temperature conditions, the pathogen mycelia could grow at 5–35 °C, and the optimum temperature was 25–30 °C. The colony diameter was the largest at 28 °C, with an average diameter of 85.00 mm, which was significantly higher than under other temperature conditions (Figure 5C). The mycelia of ABL2 could grow at pH 3–10, and the average colony diameter was the largest, with 82.38 mm at pH 7, which was significantly different from the other treatments (Figure 5D). In conclusion, the optimum growth temperature of *A. tenuissima* ABL2 was 28 °C and the optimum pH was 7. 

### 3.6. Botanical Fungicides Sensitivity of A. tenuissima ABL2

#### 3.6.1. Inhibitory Effects of Different Botanical Fungicides on the Mycelial Growth of *A. tenuissima* ABL2

**Figure 6:**
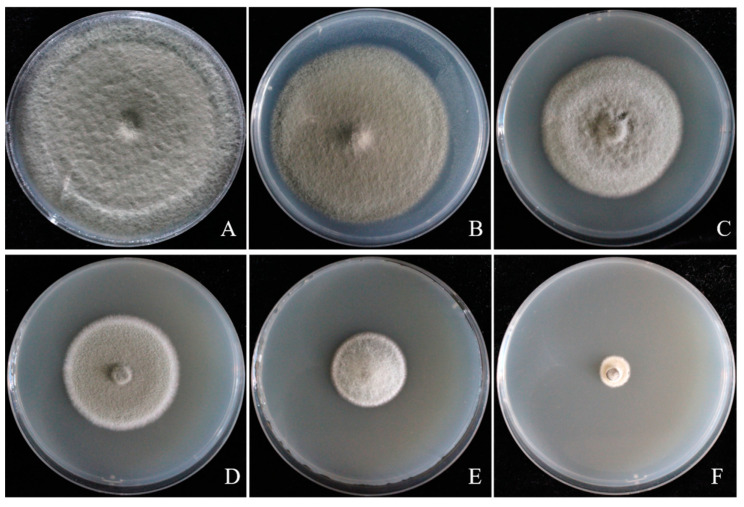
Inhibitory effect of eugenol with different concentrations on *Alternaria tenuissima* ABL2. (**A**) CK; (**B**) 0.4 μg/mL; (**C**) 0.8 μg/mL; (**D**) 1.6 μg/mL; (**E**) 3.2 μg/mL; (**F**) 6.4 μg/mL.

Different botanical fungicides had significant antifungal activity on *A. tenuissima* ABL2. Eugenol and carvacrol showed significantly higher inhibitory activity against ABL2 than the chemical fungicide pyraclostrobin + dithianon esters and other botanical fungicides, among which eugenol showed the best inhibitory effect, with an EC_50_ value of 0.826 μg/mL, followed by matrine with an EC_50_ of 4.383 μg/mL, and pyraclostrobin + dithianon with an EC_50_ of 16.702 µg/mL. The inhibition effects of eugenol on mycelial growth of ABL2 at a series of concentrations of 0.4, 0.8, 1.6, 3.2, and 6.4 μg/mL are shown in Figure 6. Ethylicin and berberine showed lower inhibitory activity against ABL2 than the chemical fungicide pyraclostrobin + dithianon, in which carvacrol and ethylicin’s EC_50_ values were 13.655 µg/mL and 20.948 μg/mL, respectively, and berberine showed a weak inhibitory effect with an EC_50_ value of 41.767 μg/mL (Table 3).

#### 3.6.2. Inhibitory Effects of Different Botanical Fungicides on Spore Germination of the *A. tenuissima* ABL2

Spore germination of *A. tenuissima* ABL2 was inhibited by different concentrations of the fungicides. An increase in the fungicide concentrations increased the inhibition rate. Among the five fungicides, eugenol and matrine showed the highest inhibition rates, with EC_50_ values of 0.755 μg/mL and 6.352 μg/mL, respectively, as well as pyraclostrobin + dithianon with an EC_50_ value of 15.366 μg/mL. Carvacrol and ethylicin had inhibition effects on the spore germination of ABL2, with EC_50_ values of 20.109 μg/mL and 27.259 μg/mL. Berberine also showed an inhibition effect, with an EC_50_ value of 40.096 μg/mL (Table 4).

### 3.7. Protective and Curative Activity of Eugenol

**Figure 7:**
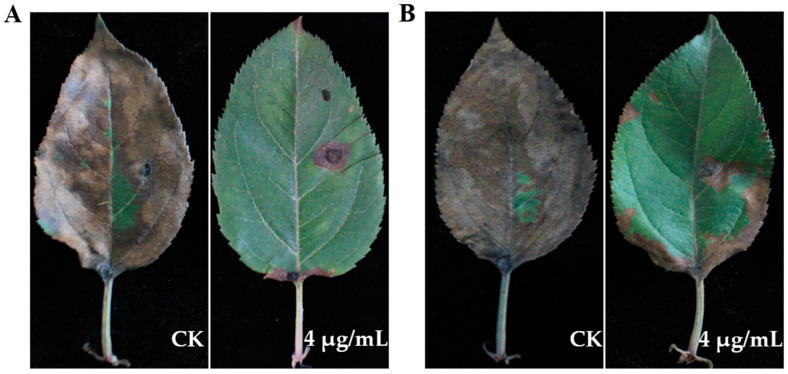
Protective and curative activity of eugenol against *A. tenuissima* ABL2 on apple leaves. (**A**) Protective activity; (**B**) Curative activity.

The protective and curative activity of eugenol against *A. tenuissima* ABL2 on detached apple leaves was determined. The results showed that eugenol exhibited both protective and curative activity on apple leaves. In protective activity assay, with application of eugenol at 4 μg/mL, the disease severity only reached 11.70%, and the control efficacy reached 86.85%. In the curative activity assay, after application of eugenol the disease severity was 21.03%, and the control efficacy reached 76.94% (Table 5, Figure 7). In the same concentration, the protective activity of eugenol against *A. tenuissima* ABL2 was better than its curative activity.

## 4. Discussion

In the current study, the *Alternaria* spp. responsible for leaf blight of apple was identified using both molecular and morphological methods. Our study provided evidence that the leaf blight pathogens on apple leaves in Gansu Province of China were all associated with *Alternaria*. From our findings, ABL2 was identified as the main pathogen of apple leaf blight following a pathogenicity test. Morphological and phylogenetic analysis of the ITS, OPA10-2, *Alta-1*, and *endoPG* gene sequences showed that ABL2 was *A. tenuissima*. Conidial length and width were two morphological characteristics used for species differentiation [40]. The conidia sizes observed in our study matched the dimensions (4.8 to 15.7 μm in width and 7.1 to 31.8 μm in length) previously described by Zhang, et al. [41]. A clear separation of species was obtained in the combined phylogeny based on the OPA10-2, *Alta-1*, and *endoPG* sequences. For *Alternaria*-caused leaf spot disease of apple trees, *A. alternata* Apple Pathotype has been identified as the causal agent of Alternaria leaf spot disease in Japan, China, and the United State [22]. Compared with *A. alternata*, *A. tenuissima* occurs more rapidly and severely, causing a large proportion of apple leaves to wither and drop in a short time, which is why it is called Alternaria leaf blight. It has been reported that *A. tenuissima* could infect a variety of plants and cause the occurrence of leaf blight, such as *Citrullus lanatus* [42], *Helianthus annuus* [43], *Zea mays* [44], *Saccharum officinarum* [45], etc. Harteveld, et al. [14] found that leaf spot disease and fruit spot disease of apples caused by *Alternaria* species occur in apple orchards in Australia, and that the *Alternaria* species causing these two diseases are mainly *A. arborescens* (47%), *A. alternata*/*A. tenuissima* (14%), and *A. tenuissima*/*A. mali* (6%), but leaf blight disease of apples caused by *A. tenuissima* was first discovered in China.

Nutrient media with different carbon and nitrogen sources, pH, and temperature can affect the mycelial growth and virulence of pathogenic fungi [17,46], so it is helpful to clarify the biological characteristics of pathogens to better understand the field incidence of diseases. In this study, the optimal carbon, nitrogen source, temperature and pH for *A. tenuissima* were soluble starch, soybean flour, 28 °C and 7. The results of the biological characterization of *A. tenuissima* showed that the disease is more likely to outbreak in warm, humid seasons, which is consistent with the law of pathogenesis of apple blight in our study.

Disease control strategies that have been investigated as alternatives to currently used fungicides to control plant diseases caused by *A. tenuissima* generally include the use of new fungicides, antagonistic microorganisms [47], and plant extracts [48,49]. Although application of fungicide is still the main method for control of apple tree diseases, continuous and extensive use of a single chemical may lead to undesirable effects such as environmental pollution, residue toxicity, and the risk of fungicide resistance. Botanical fungicides are becoming more popular due to their specific antifungal activity, ease of degradation, and safety to humans. In recent years, several studies have focused on screening plant extracts to develop new antifungal botanical fungicides that can be used to control apple tree diseases [50,51]. In this study, eugenol showed excellent ability to inhibit *Alternaria* hyphal growth and spore germination compared to the synthetic control fungicide. However, indoor research conditions are relatively stable, while uncontrollable environmental factors may exist in the field. Therefore, whether this agent can be used effectively and stably in the field needs further experimental investigation. To the best of our knowledge, our study provides the first report on the identification of *A. tenuissima* as the causal agent of apple leaf blight in Gansu Province, China.

## 5. Conclusions

The current study demonstrated that apple leaf blight in apple-producing regions in Gansu Province, China, is associated with isolates of *A. tenuissima*. Carbon sources, nitrogen sources, temperature, and pH were experimentally confirmed to be essential for the growth of *A. tenuissima* ABL2. Furthermore, our results showed that eugenol could be considered as a beneficial biocontrol agent in controlling apple leaf blight. Future research will focus on whether eugenol has the ability to protect against *A. tenuissima* throughout the whole disease cycle under different environmental conditions in the field.

## Figures and Tables

**Table 1:** Characteristics of primer pairs used in this study for PCR assay.

Locus	Primers	Sequence (5′-3′)	Annealing Temperature and Duration	Reference
Internal transcribed spacer	ITS1	TCCGTAGGTGAACCTGCGG	54 °C, 30 s	[32]
	ITS4	TCCTCCGCTTATTGATATGC		
Anonymous noncoding region	OPA10-2R	GATTCGCAGCAGGGAAACTA	62 °C, 30 s	[33]
	OPA10-2L	TCGCAGTAAGACACATTCTACG		
Alternaria major allergen 1	*Alta1-R*	ACGAGGGTGAYGTAGGCGTC	57 °C, 45 s	[34]
	*Alta1-F*	ATGCAGTTCACCACCATCGC		
Endopolygalacturonase	*EndoPG3*	TACCATGGTTCTTTCCGA	62 °C, 30 s	[14]
	*EndoPG2b*	GAGAATTCRCARTCRTCYTGRTT		

**Table 2:** Different fungicides and diluted concentrations.

Fungicide	Formulation	Effective Components (%)	Fungicide Concentrations (μg/mL)				
Matrine	AS	1.3	2	4	6	8	10
Eugenol	SL	0.3	0.4	0.8	1.6	3.2	6.4
Carvacrol	AS	5	10	15	20	25	30
Ethylicin	EC	80	15	20	25	30	35
Berberine	AS	0.5	30	35	40	45	50
Dithianon + Pyraclostrobin	SC	25	10	15	20	25	30

AS = aqueous solution; SC = suspension concentrate; SL = soluble concentrate; EC = emulsifiable concentrate.

**Table 3:** Inhibitory effects of different botanical fungicides on the colony growth of *Alternaria tenuissima* ABL2.

Fungicide	Concentrations (μg/mL)	Inhibitory Rate (%)	Regression Equation	Correlation Coefficient (r)	EC_50_ (μg/mL)	95% Confidence Interval
1.3% Matrine	2	30.15 ± 1.72	y = 1.7360x + 3.8860	0.9808	4.383	3.834~5.010
	4	44.38 ± 0.95				
	6	56.75 ± 1.24				
	8	65.16 ± 1.21				
	10	77.46 ± 1.20				
0.3% Eugenol	0.4	34.61 ± 1.48	y = 1.4356x + 5.1191	0.9955	0.826	0.727~0.938
	0.8	48.50 ± 2.61				
	1.6	63.99 ± 2.02				
	3.2	78.23 ± 1.40				
	6.4	91.25 ± 2.61				
5% Carvacrol	10	34.75 ± 2.93	y = 3.7036x + 0.7941	0.9802	13.665	12.196~15.311
	15	53.15 ± 1.03				
	20	68.34 ± 3.77				
	25	81.04 ± 2.82				
	30	92.70 ± 0.64				
20% Ethylicin	15	34.82 ± 2.57	y = 2.9386x + 1.1176	0.9927	20.948	20.023~21.917
	20	47.14 ± 1.67				
	25	56.93 ± 1.76				
	30	66.10 ± 1.57				
	35	76.59 ± 3.00				
0.5% Berberine	30	17.71 ± 1.44	y = 6.2843x + 5.1859	0.9990	41.767	41.364~42.175
	35	32.71 ± 1.30				
	40	45.71 ± 1.57				
	45	57.09 ± 1.81				
	50	68.96 ± 0.95				
25% Pyraclostrobin + dithianon	10	37.79 ± 1.70	y = 1.6381x + 2.9770	0.9864	16.702	15.481~18.020
	15	44.25 ± 1.03				
	20	53.56 ± 1.26				
	25	62.08 ± 1.45				
	30	67.34 ± 2.76				

**Table 4:** Effects of different botanical fungicides on the spore germination of *Alternaria tenuissima* ABL2.

Fungicide	Concentrations (μg/mL)	Spore Germination Inhibition Rate (%)	Regression Equation	Correlation Coefficient (r)	EC_50_ (μg/mL)	95% Confidence Interval
1.3% Matrine	2	24.66 ± 1.58	y = 1.5826x + 3.7293	0.9610	6.352	5.221~7.730
	4	33.12 ± 1.66				
	6	43.25 ± 1.81				
	8	56.23 ± 2.66				
	10	67.50 ± 0.63				
0.3% Eugenol	0.4	37.71 ± 2.38	y =1.3859x + 5.1689	0.9902	0.755	0.620~0.920
	0.8	51.08 ± 3.29				
	1.6	64.59 ± 1.99				
	3.2	77.87 ± 2.26				
	6.4	91.96 ± 2.57				
5% Carvacrol	10	26.67 ± 2.19	y = 2.2011x + 2.1312	0.9907	20.109	18.910~21.383
	15	37.65 ± 1.66				
	20	48.13 ± 3.83				
	25	56.46 ± 1.57				
	30	67.71 ± 0.95				
20% Ethylicin	15	27.92 ± 1.30	y = 2.4335x + 1.5067	0.9830	27.259	25.443~29.204
	20	36.67 ± 1.57				
	25	43.54 ± 2.34				
	30	52.08 ± 2.42				
	35	63.75 ± 1.65				
0.5% Berberine	30	22.92 ± 1.40	y = 6.0534x + 4.7042	0.9957	40.096	39.339~40.867
	35	36.88 ± 2.25				
	40	46.96 ± 0.91				
	45	60.83 ± 2.36				
	50	73.75 ± 1.25				
25% Dithianon + pyraclostrobin	10	32.46 ± 2.98	y =2.6573x + 1.8469	0.9913	15.366	14.379~16.401
	15	48.71 ± 1.58				
	20	58.54 ± 4.38				
	25	70.17 ± 3.43				
	30	80.42 ± 2.61				

**Table 5:** Protective and curative activity of eugenol against *A. tenuissima* ABL2 on leaves of apple.

	Disease Severity (%)		Control Efficacy (%)
	CK	Eugenol	
Protective activity	88.26 ± 3.08	11.70 ± 4.71	86.85 ± 4.86
Curative activity	91.02 ± 3.66	21.03 ± 3.01	76.94 ± 2.58

## Data Availability

The data in this study are available on request from the corresponding author/first author.

## Supplementary Materials

The following supporting information can be downloaded at: https://www.mdpi.com/article/10.3390/jof10040255/s1, Table S1: Reference sequences, species, sources, and GenBank accession numbers used for phylogenetic analysis in the study.
