# Supplementary material for: Identification the Pathogen Cause a New Apple Leaf Blight in China and Determination the Controlling Efficacy for Five Botanical Fungicides
Source: J Fungi (Basel). 2024 Mar 27;10(4):255. doi: 10.3390/jof10040255 (PMC11051572; doi:10.3390/jof10040255)
Supplement: Supplementary file 1 [file jof-10-00255-s001.zip › jof-2907939-supplementary.pdf]

Table S1. Reference sequences, species, sources, and GenBank accession numbers used for phylogenetic analysis in the study.

Species	Strain	Locality, host/substrate	GenBank accession		
			Alt a-1	*endoPG*	OPA10-2
Alternaria alternata	19-51-31	Italy, *Clematis japonica*	MT043357	MT185590	MT043355
Alternaria alternata	CBS 686.68	Sahara, desert sand	KP123859	KP124006	KP124614
Alternaria alternata	CBS 107.53	Japan, *Pyrus pyrifolia*	KP123858	KP124005	KP124613
Alternaria alstroemeriae	EGS 50.116	USA, *Alstroemeria* sp.	MN975274	MN975303	MN975323
Alternaria arborescens	EGS 39.128	USA, *Solanum lycopersicum*	MN975269	MN975299	MN975318
Alternaria gaisen	EGS 90.512	Japan, *Pyrus pyrifolia*	MN975272	MN975301	MN975320
Alternaria tenuissima	SY-4*	China, *Dioscorea zingiberensis*	MK593137	MK593136	EF503979
Alternaria tenuissima	DES512*	USA, Desert soil	JQ282240	EF504117	EF503988
Alternaria longipes	EGS 30.033	USA, *Nicotiana tabacum*	MN975273	MN975302	MN975321
Alternaria longipes	CBS 539.94	USA, *Nicotiana tabacum*	KP123987	KP124146	KP124757
Alternaria longipes	CBS 917.96	USA, *Nicotiana tabacum*	KP123988	KP124148	KP124759
Alternaria jacinthicola	CBS 878.95	Mauritius, *Arachis hypogaea*	KP123983	KP124142	KP124753
Alternaria toxicogenica	PR320	*USA, Citrus reticulata*	JQ646411	AY295027	EF504057
Alternaria arborescens	CPC 25266	Austria, *Pyrus* sp.	KP123965	KP124122	KP124732
Alternaria burnsii	CBS 107.38	India, *Cuminum cyminum*	KP123967	KP124124	KP124734
Alternaria burnsii	CBS 118817	India, *Tinospora cordifolia*	KP123971	KP124128	KP124738
Alternaria eichhorniae	CBS 489.92	India, *Eichhornia crassipes*	KP123973	KP124130	KP124740
Alternaria gossypina	CBS 104.32	Zimbabwe, *Gossypium* sp.	JQ646395	KP124135	KP124746
Alternaria gossypina	CBS 102601	Colombia, *Minneola tangelo*	KP123979	KP124138	KP124749
Alternaria iridiaustralis	CBS 118486	Australia, *Iris* sp.	KP123981	KP124140	KP124751
Alternaria tomato	CBS 103.30	Unknown, *Solanum lycopersicum*	KP123991	KP124151	KP124762
	**ABL2**	**China, *Malus pumila***	**MZ222271**	**MZ222269**	**MZ222272**

Isolate obtained in this study were marked in bold. The accession number of another fungal strain from GenBank were marked*.
